# Anti‐inflammatory effect of *Antirrhinum majus* extract in lipopolysaccharide‐stimulated RAW 264.7 macrophages

**DOI:** 10.1002/fsn3.1805

**Published:** 2020-07-23

**Authors:** Mi Jang, Inguk Hwang, Byungsoon Hwang, Gichang Kim

**Affiliations:** ^1^ National Academy of Agricultural Science Rural Development Administration Jeonju Korea

**Keywords:** anti‐inflammatory, antioxidative, *Antirrhinum majus*, cytokines, ethanol extract

## Abstract

*Antirrhinum majus* (AM) has attracted attention as a rich source of phytochemicals, which are beneficial for human health. However, the anti‐inflammatory effects of AM have not been studied scientifically. Therefore, we investigated the antioxidative properties and anti‐inflammatory effects of AM extract (AME) in lipopolysaccharide (LPS)‐stimulated RAW 264.7 macrophages. AME showed high radical‐scavenging ability. Viability of RAW 264.7 cells was not significantly altered by AME at the concentrations of 0–300 µg/ml. LPS‐induced nitric oxide (NO) production was decreased by treatment with 0–300 µg/ml AME in a concentration‐dependent manner. AME pretreatment significantly inhibited the protein expression of inducible nitric oxide synthase (iNOS) and cyclooxygenase‐2 (COX‐2) in a concentration‐dependent manner. AME also considerably inhibited the mRNA and protein expression of inflammatory cytokines, such as tumor necrosis factor‐a (TNF‐α), interleukin‐1 β (IL‐1β), and interleukin‐6 (IL‐6). These findings provide a foundation for further studies and use of AM in nutraceuticals.

## Graphical abstract

Antirrhinum majus extract showed strong inhibition of inflammatory cytokines (TNF‐alpha, IL) and an protein expression of iNOS and COX‐2. Also, it show high radical‐scavenging ability. These findings provide a foundation for further studied and use as nutraceuticals.

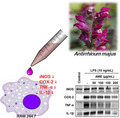

## 1 INTRODUCTION

Inflammatory response refers to a response in which the immune cells secrete various inflammatory mediators to protect the body in the event of trauma caused by an external physical or chemical stimulus or introduction of foreign substances such as bacteria or virus (Dinarello, 2010; Olefsky & Glass, 2010). Sustained inflammatory response can lead to an excessive secretion of inflammatory mediators, which cause various adult diseases, including obesity, diabetes, and hypertension (Charo & Taub, 2011; Deans & Sattar, 2006; Ohashi, Shibata, Murohara, & Ouchi, 2014; Wojdasiewicz, Poniatowski, & Szukiewicz, 2014; Wong, Spence, Lamki, Freeman, & McDonald, 1986). Macrophages, which are distributed throughout the body tissues, play a key role in this inflammatory response. They not only possess the ability to defend against pathogens and cancer cells but are also activated by various stimuli or cytokines secreted by immune cells; this enables macrophages to secrete various physiologically active substances and pro‐inflammatory cytokines, such as nitric oxide (NO), tumor necrosis factor (TNF‐α), interleukin (IL)‐1β, and IL‐6, to maximize the immune response (Scheller, Chalaris, Schmidt‐Arras, & Rose‐John, 2011; Valledor, Comalada, Santamaría‐Babi, Lloberas, & Celada, 2010). Also, MCP‐1, a potent chemical factor in the CC‐chemotactic family, is caused by the stimulation of LPS in macrophages. It is one of the key chemokines the regulate infiltration and migration of monocytes and macrophages to sites of active inflammation (Jamie et al., 2012; Satish, Sergey, Shohreh, & Bassel, 2009). Inflammation within the body causes the production of a large amount of inflammatory factors, such as NO and prostaglandin E_2_ (PEG_2_), by inducible nitric oxide synthase (iNOS) and cyclooxygenase‐2 (COX‐2). In the inflamed state, excessive amount of NO produced by iNOS not only facilitates inflammatory response but also exacerbates the inflammation by facilitating the biosynthesis of inflammatory mediators (Choi, Kim, & Lee, 2014; Fiorucci et al., 2004). In other words, the expression of iNOS and COX‐2 and the production of NO and PEG_2_ serve as typical inflammatory factors of immune cells.

Moreover, another factor that can induce an inflammatory response is the oxidative stress generated inside the body. The biochemical reactions of normal cells, along with various environmental pollutants and chemicals, increase oxidative stress in the body, which causes cellular damage, including cell membrane damage and protein degradation. Furthermore, because it is linked to inflammatory response, oxidative stress can also induce chronic inflammatory response that leads to DNA damage and various diseases such as cancer, heart disease, arteriosclerosis, Alzheimer's disease, and Parkinson's disease (Halliwell & Gutteridge, 1984, 2015; Hybertson, Gao, Bose, & McCord, 2011; Reuter, Gupta, Chaturvedi, & Aggarwal, 2010; Uttara, Singh, Zamboni, & Mahajan, 2009; Valko et al., 2007). Accordingly, chronic inflammatory response can be reduced by decreasing oxidative stress via consumption of ingredients with excellent antioxidative effect (Di Penta et al., 2013; Elmarakby & Sullivan, 2012).

*Antirrhinum majus* (AM), which belongs to the Scrophulariaceae family, is a perennial plant that originates from Africa and Southern Europe. AM was so named because its flower resembles the mouth of a goldfish. Its seeds, leaves, and flowers are used as herbal medicines, and AM is known to have anti‐inflammatory, analgesic, and diuretic effects; however, scientific studies on the physiological activity of AM are still lacking. Therefore, the present study aimed to identify the functionality of AM extract (AME) to explore whether it can be used as a basic ingredient in the development of agents for preventing and treating inflammatory diseases. We investigated the in vitro antioxidative activities of AME, along with its inhibitory effects on the production of NO, iNOS, COX‐2, and inflammatory cytokines in lipopolysaccharide (LPS)‐induced RAW 264.7 cells.

## 2 MATERIALS AND METHODS

### 2.1 AM sample and extract preparation

*Antirrhinum majus* used in the present study was purchased from Five Mountain Herb Farm in 2016. The purchased AM was washed twice with water, and the moisture was removed. Subsequently, 100 g AM was submersed in 95% ethanol and ground with a homogenizer (IKA) and then incubated in the dark for 24 hr at 25°C. AM‐ethanol extract was filtered using a filter paper (NO. 2; Whatman), followed by decompression concentration with a rotary vacuum evaporator (R‐205; Buchi) at 37°C. The concentrated AM extract (AME) was freeze‐dried, powdered, and stored at −20°C for use in further experiments.

### 2.2 Measurement of total polyphenol content

Total polyphenol content from the AME was measured via a method modified from the Folin–Denis method (Singleton & Rossi, 1965). Here, 500 μl of the extract was reacted with 500 μl of 0.5 N Folin–Ciocalteu's phenol reagent in the dark for 3 min, followed by addition of 1.5 ml of 2% Na_2_CO_3_ and incubation for 30 min in the dark at room temperature. The samples were dispensed into a 96‐well plate, and the absorbance at 760 nm was measured using a microplate reader (Infinite M200 PRO; Tecan). TPC was calculated by obtaining the slope and intercept values from the standard calibration curve constructed using the absorbance values based on gallic acid solutions at four different concentrations (1, 10, 100, and 1,000 μg/ml) and then substituting the absorbance value with that of each sample.

### 2.3 Measurement of total flavonoid content

Total flavonoid content was measured via the method described by Moreno, Isla, Sampietro, and Vattuone (2000). Briefly, 1 ml of the extract was mixed with 1 ml of 2% AlCl_3_ and incubated in the dark for 30 min, followed by measuring the absorbance at 430 nm using a microplate reader. TFC was calculated by obtaining the slope and intercept values from the standard calibration curve constructed using the absorbance values based on quercetin solutions at four different concentrations (1, 10, 100, and 1,000 μg/ml) and then substituting the absorbance value with that of each sample.

### 2.4 Measurement of DPPH and ABTS radical‐scavenging ability

The Blois method was used to measure the electron donating ability of AME samples with respect to 1,1‐diphenyl‐2‐picrylhydrazyl (DPPH; Sigma‐Aldrich Co.) by using (Blois, 1958). The AME samples were diluted to concentrations of 1, 2, 5, and 10 μg/ml. Then, 1 ml of 0.15 mM DPPH solution was added to 1 ml of each diluted sample, the mixtures were incubated for 30 min in the dark, and the absorbance at 517 nm was measured. The 2,2'‐azinobis(3‐ethylbenzothiazoline‐6‐sulfonate) (ABTS) radical‐scavenging ability was measured using the method described by Pellegrini et al (Re et al., 1999). After thoroughly mixing 7 mM ABTS (Sigma‐Aldrich Co.) and 2.45 mM K_2_S_2_O_8_ (potassium persulfate; Sigma‐Aldrich, Co.) solutions, the mixture was stored for 12–16 hr in the dark. The ABTS solution was used by precalibrating its OD value at 734 nm to be 0.7–1.1. After mixing 100 μl of ABTS solution with 100 μl of samples diluted to varying concentrations, the mixtures were dispensed to a 96‐well plate and allowed to react for 30 min in the dark, after which, absorbance at 732 nm was measured. Ascorbic acid was used as the positive control for DPPH and ABTS radical‐scavenging ability.

### 2.5 Cell culture

RAW 264.7 macrophages were obtained from the American Type Culture Collection (ATCC). RAW 264.7 macrophages were cultured in 37°C, 5% CO_2_ incubator with Dulbecco's modified Eagle's medium (DMEM; Sigma‐Aldrich Co.) containing 10% fetal bovine serum (FBS; Gibco), and 1% Penicillin‐Streptomycin solution (Sigma‐Aldrich Co.).

### 2.6 Cytotoxicity

Cytotoxicity in RAW 264.7 macrophages was tested via 3‐(4,5‐dimethylthiazole‐2‐yl)‐2,5‐diphenyl‐tetrazolium bromide (MTT; Sigma‐Aldrich Co.) assay. Cells were seeded into a 96‐well plate, with 1 × 10^3^ cells/well, and cultured in 37°C, 5% CO_2_ incubator for 24 hr. After treating the cells with varying concentrations of AME, the cells were cultured again for 24 hr under the same conditions. Next, 50 μl of 5 mg/ml MTT solution was added to each well, and the cells were cultured for 4 hr. Then, the medium was removed, the cells were washed twice with phosphate‐buffered saline (PBS), and 200 μl of dimethyl sulfoxide (DMSO; Sigma‐Aldrich Co.) was added to each well. Absorbance at 540 nm was measured using a microplate reader.

### 2.7 Measurement of NO concentration

RAW 264.7 macrophages were cultured in 96‐well plates, under the conditions mentioned above. After pretreating with varying concentrations of AME for 4 hr, the cells were treated with LPS (10 ng/ml) and cultured for 24 hr. The cell culture broth was retrieved, mixed with Griess reagent at a 1:1 ratio, and incubated in the dark at room temperature for 10 min. Absorbance at 540 nm was measured using a microplate reader.

### 2.8 Western blotting

Raw 264.7 macrophages were seeded in a 6‐well plate (2 × 10^5^ cells/well) and cultured for 24 hr. After pretreating with varying concentrations of AME for 4 hr, the cells were treated with LPS (10 ng/ml) and cultured for another 24 hr. The medium was removed; the cells were washed with PBS and then centrifuged at 5,000 *g* for 3 min. The pellet thus obtained was resuspended in lysis buffer (GenDEPOT) and incubated for 30 min on ice, followed by sonication for 60 s. After confirming that the lysis buffer had lost its viscosity, the mixture was centrifuged at 10,000 *g* for 10 min to collect the supernatant. Proteins in the supernatant were quantified using BSA assay kit (Thermo Fisher Scientific Inc.). Quantified protein was mixed in 4× sample buffer and inactivated for 10 min at 100°C, followed by electrophoresis with 12% SDS poly‐acrylamide gel. The isolated proteins were transblotted onto polyvinylidene difluoride (PVDF) membrane, which was then blocked by 2‐hr incubation with 5% skim milk. The membrane was incubated with primary antibodies against iNOS (1:3,000, sc7271; Santa Cruz Biotechnology), COX‐2 (1:5,000, ab15191; Abcam), IL‐1β (1:3,000, ab9722; Abcam), TNF‐α (1:3,000, ab9739; Abcam), and GAPDH (1:3,000, ab9485; Abcam) for 24 hr at 4°C, and washed 3 times for 10 min with Tris‐buffered saline Tween‐20 (TBST). Secondary antibodies were diluted to a ratio of 1:5,000 and allowed bind for 2 hr at room temperature. After washing 3 times for 10 min with TBST buffer, the samples were treated with enhanced chemiluminescence (ECL) solution and imaged using the ChemiDoc system (Bio‐Rad).

### 2.9 Total RNA extraction and RT‐qPCR

RAW 264.7 macrophages, cultured as described in the earlier sections, were washed twice with PBS. Total RNA was isolated using the NucleoSpin^®^ RNA Plus kit (Macherey‐Nagel) and quantified using NanoDrop One spectrophotometer (Thermo Fisher Scientific Inc.).Then, 1 μg of total RNA was reverse‐transcribed into cDNA using the High Capacity cDNA Reverse Transcription Kit (Thermo Fisher Scientific Inc.). Real‐time PCR was performed using 1 μl of the synthesized cDNA, together with 1 μl of primer, 10 μl of SYBR master mix, and 8 μl of RNA‐free water. The mRNA expression of all genes tested is normalized to the RPS3 expression. Gene‐specific information of the genes analyzed is provided in Table 1.

**TABLE 1:** Sequences of primers used for RT‐qPCR

Primer name	Sequence
*iNOS*	
Forward	AATCTTGGAGCGAGTTGTGG
Reverse	CAGGAAGTAGGTGAGGGCTTG
*COX‐2*	
Forward	ACTCACTCAGTTTGTTGAGTCATT
Reverse	TTTGATTAGTACTGTAGGGTTAATG
*TNF‐α*	
Forward	GCCACCACGCTCTTCTGCCT
Reverse	GGCTGATGGTGTGGGTGAGG
*MCP‐1*	
Forward	TCTGGACCCATTCCTTCTTG
Reverse	AGGTCCCTGTCATGCTTCTG
*IL‐6*	
Forward	CCAGAGATACAAAGAAATGATGG
Reverse	ACTCCAGAAGACCAGAGGAAAT
*IL‐1β*	
Forward	AAATACCTGTGGCCTTGGGC
Reverse	CTTGGGATCCACACTCTCCAG
*RPS3*	
Forward	ATCAGAGAGTTGACCGCAGTTG
Reverse	AATGAACCGAAGCACACCATAG

### 2.10 Statistical analysis

Significance between groups was tested using GraphPad Prism version 5.02 (GraphPad Software Inc.), and the results were expressed as mean ± *SEM*. Significance of differences between the mean values for two groups was assessed using *t* test. For all statistical significance, differences with *p*‐values <.05 were considered significant.

## 3 RESULTS AND DISCUSSION

### 3.1 High TPC and TFC in AME

Phenols are the most abundant of the various physiologically active substances present in plants (Manach, Scalbert, Morand, Rémésy, & Jiménez, 2004). Polyphenolic compounds are largely divided into flavonoids and tannins. Of the two, flavonoids belong to the largest subgroup of polyphenols synthesized by plants. The total polyphenols present in AM were measured in terms of gallic acid equivalents, whereas the total flavonoids were measured in terms of quercetin equivalents. The AME showed a high gallic acid and quercetin equivalent contents of 31.65 and 0.28 mg/g, respectively (Table 2). The high content of polyphenols, which are antioxidative substances that convert active oxygen in the body into harmless substances, in AM confirms that AM would have excellent antioxidative effects.

**TABLE 2:** Total polyphenol and flavonoid contents of *Antirrhinum majus* extracts

Sample	Total polyphenol content	Total flavonoid content
	(mg GAE/g)	(mg QE/g)
95% ethanol extract of *Antirrhinum majus*	31.65 ± 0.63	0.28 ± 0.02

Abbreviations: GAE, gallic acid equivalent; QE, quercetin equivalent.

### 3.2 Radical‐scavenging activities of AME

**FIGURE 1:**
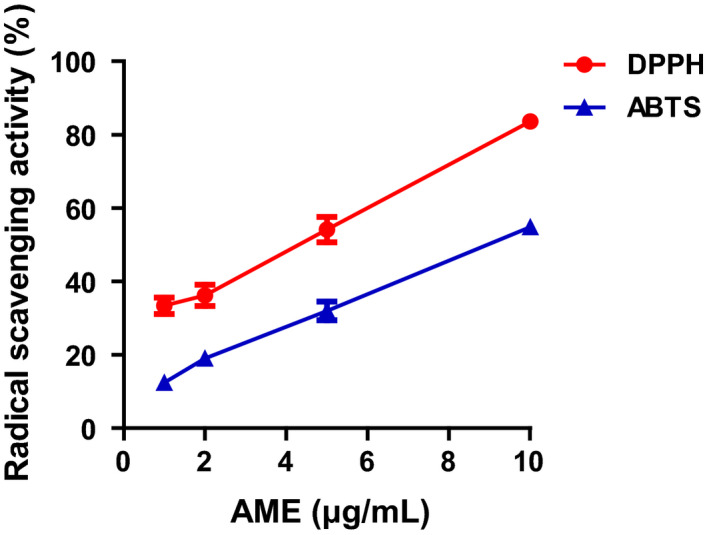
Antioxidant activity of *Antirrhinum majus* extract (AME). DPPH radical‐scavenging activity was measured for a mixture containing 0.15 mM DPPH and different concentrations of AME. For the ABTS assay, 7 mM ABTS solution was incubated with 2.45 mM potassium persulfate; and the ABTS radical‐scavenging ability of a mixture of 100 μl of this solution and different concentrations of AME was measured

DPPH and ABTS assays were performed to analyze the antioxidative properties of AME in terms of radical‐scavenging ability. DPPH is a free radical with a unique color. However, when free radical DPPH reacts with an antioxidant, the free radicals are scavenged by the antioxidative action of the sample, thereby resulting in a color change from purple to yellow. The method based on this principle is widely used for measuring antioxidative activities (Bondet, Brand‐Williams, & Berset, 1997). The DPPH radical‐scavenging ability of AME increased as the concentration of AME increased (Figure 1). ABTS radical‐scavenging ability is measured on the basis of the principle that antioxidants within the sample scavenge ABTS free radicals produced by the reaction between the ABTS reagent and potassium persulfate, thereby resulting in the loss of cyan color. Similar to the results of the DPPH assay, the ABTS radical‐scavenging ability of AME increased as the concentration of AME increased. Both DPPH and ABTS radical‐scavenging abilities were the highest at the concentration of 10 μg/ml. This confirms that AME has high antioxidative activities.

### 3.3 Cytotoxicity assessment in RAW 264.7 cells

**FIGURE 2:**
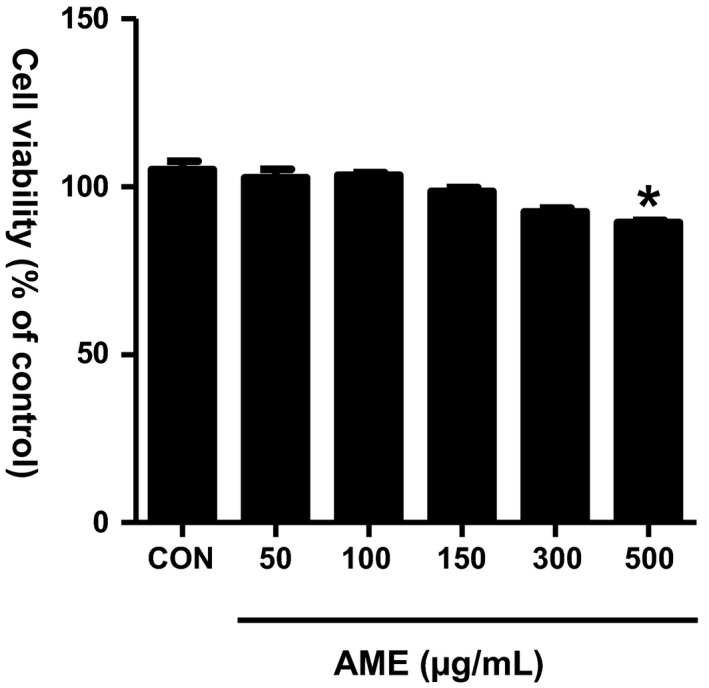
Cytotoxicity of *Antirrhinum majus* extract (AME) in RAW 264.7 cells. RAW 264.7 cells were incubated for 24 hr in the presence or absence of AME at the indicated concentration. Cell viability was evaluated by the MTT assay. Data represent the mean ± *SEM* of triplicate determinations from three separate experiments. *p < .05 versus CON

MTT assay was used to assess the cytotoxicity of AME in RAW 264.7 macrophages (Figure 2). Sample concentrations with cell viability of ≥90% relative to the control group were considered to be safe, non‐toxic concentrations. Cell viability measurement after treatment with 50, 100, 150, 300, and 500 μg/ml of AME showed cell viability of ≥90% up to the concentration of 300 μg/ml, revealing no cytotoxicity, whereas 500 μg/ml AME showed cell viability of <90%. On the basis of these results, the 50, 100, 150, and 300 μg/ml AME samples were selected for further experiments.

### 3.4 Inhibitory effects of AME on NO production

**FIGURE 3:**
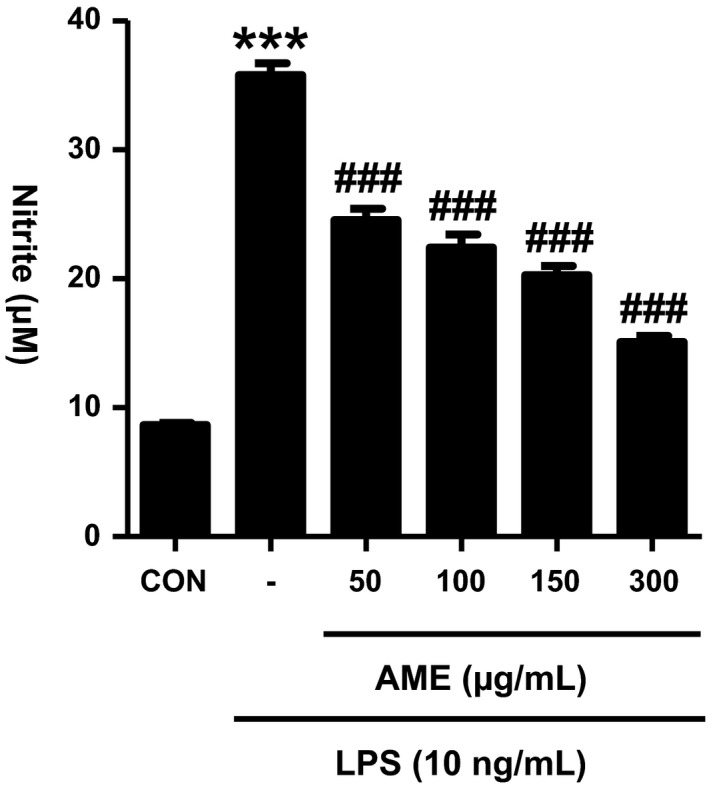
Effect of *Antirrhinum majus* extract (AME) on nitrite production in RAW 264.7 cells. After pretreatment with the indicated concentration of AME for 4 hr, RAW 264.7 cells were treated with 10 ng/ml lipopolysaccharide (LPS) for 24 hr. Nitrite levels in the cell media were measured spectrophotometrically using Griess reagent. Data are provided as mean ± *SEM*. ***p < .01 versus CON; ^###^ *p* < .05 versus LPS treatment only

Lipopolysaccharide are extracellular components of gram‐negative bacteria and they act as powerful stimuli for various cells such as monocytes and macrophages. In particular, when macrophages are activated after stimulation by LPS, they produce and release inflammatory mediators such as NO via regulation of pro‐inflammatory factors (Lin, Juan, Shen, Hsu, & Chen, 2003; Xu et al., 2014). Therefore, in the present study, we examined the effects of AME on the production of NO, which is one of the active oxygen species and is known to play a key role in inflammation induction. The amount of NO produced was measured in terms of NO_2_^−^ content of the cell culture broth by using the Griess reagent. Based on the results obtained, the inhibitory effects of AME against NO production by LPS‐stimulated RAW 264.7 cells were measured. NO production after LPS stimulation increased to 35.8 μM in the untreated cells. This LPS‐induced increase in NO was significantly inhibited by AME treatment in a concentration‐dependent manner (Figure 3). In particular, excellent inhibitory effect of ≥50% on NO production was found at the AME concentration of 300 μg/ml.

### 3.5 Anti‐inflammatory effects of AME in RAW 264.7 cells

**FIGURE 4:**
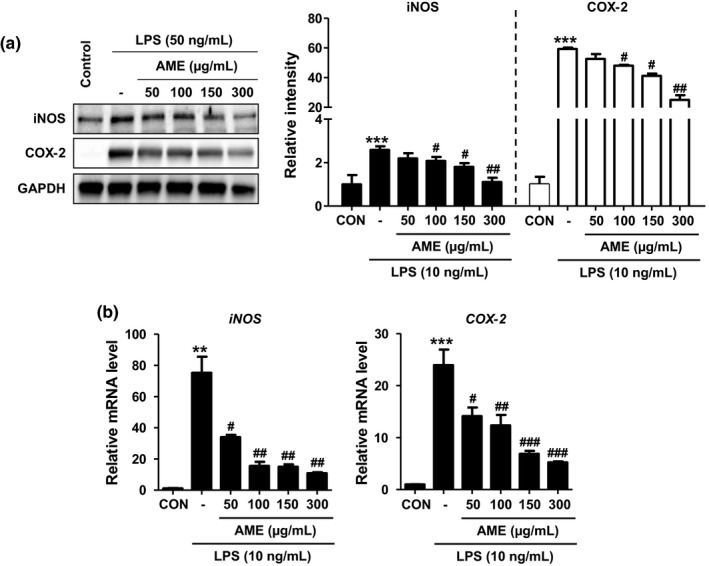
Inhibitory effects of *Antirrhinum majus* extract (AME) on lipopolysaccharide (LPS)‐induced iNOS and COX‐2 expression. Cells were treated with AME for 4 hr followed by LPS treatment (10 ng/ml) for 24 hr. (a) Concentration‐dependent effect of AME on the expression of iNOS and COX‐2. Representative Western blot images and quantification data are shown. (b) mRNA expression of inflammatory genes. Cells were treated with agents for 24 hr, and RT‐qPCR was performed. Data are provided as mean ± *SEM*. **p < .01, ***p < .01 versus CON; ^#^ *p* < .05, ^##^ *p* < .05, ^###^ *p* < .05 versus LPS treatment only

Nitric oxide, a mediator of inflammatory response, is biosynthesized by the enzymes NOS and COX. Therefore, we next performed a western blot analysis to determine whether AME also inhibits the protein expression of iNOS and COX‐2. After pretreating Raw 264.7 cells with LPS (10 ng/ml), the cells were treated with AME and cultured for 24 hr to observe expression of iNOS and COX‐2. The group that was not treated with LPS showed no iNOS and COX‐2 expression, whereas the LPS‐treated group treated showed increased expression of iNOS and COX‐2, compared to that observed in the control group. However, AME treatment showed strong inhibition of iNOS and COX‐2 expression in a concentration‐dependent manner (Figure 4a). Next, the inhibition of *iNOS* and *COX‐2* mRNA expression was studied in AME‐treated RAW 264.7 cells. The results were similar to those obtained for experiments studying the expression of iNOS and COX‐2 proteins; the AME‐treated group showed inhibitory effects on mRNA expression, compared to the mRNA expression observed in the control group that was treated with LPS only (Figure 4b). These findings show that AME has strong inhibitory effects on NO production and early‐inflammatory factor production and that NO production is inhibited via inhibition of iNOS expression.

### 3.6 Inhibitory effects of AME on pro‐inflammatory cytokine production

**FIGURE 5:**
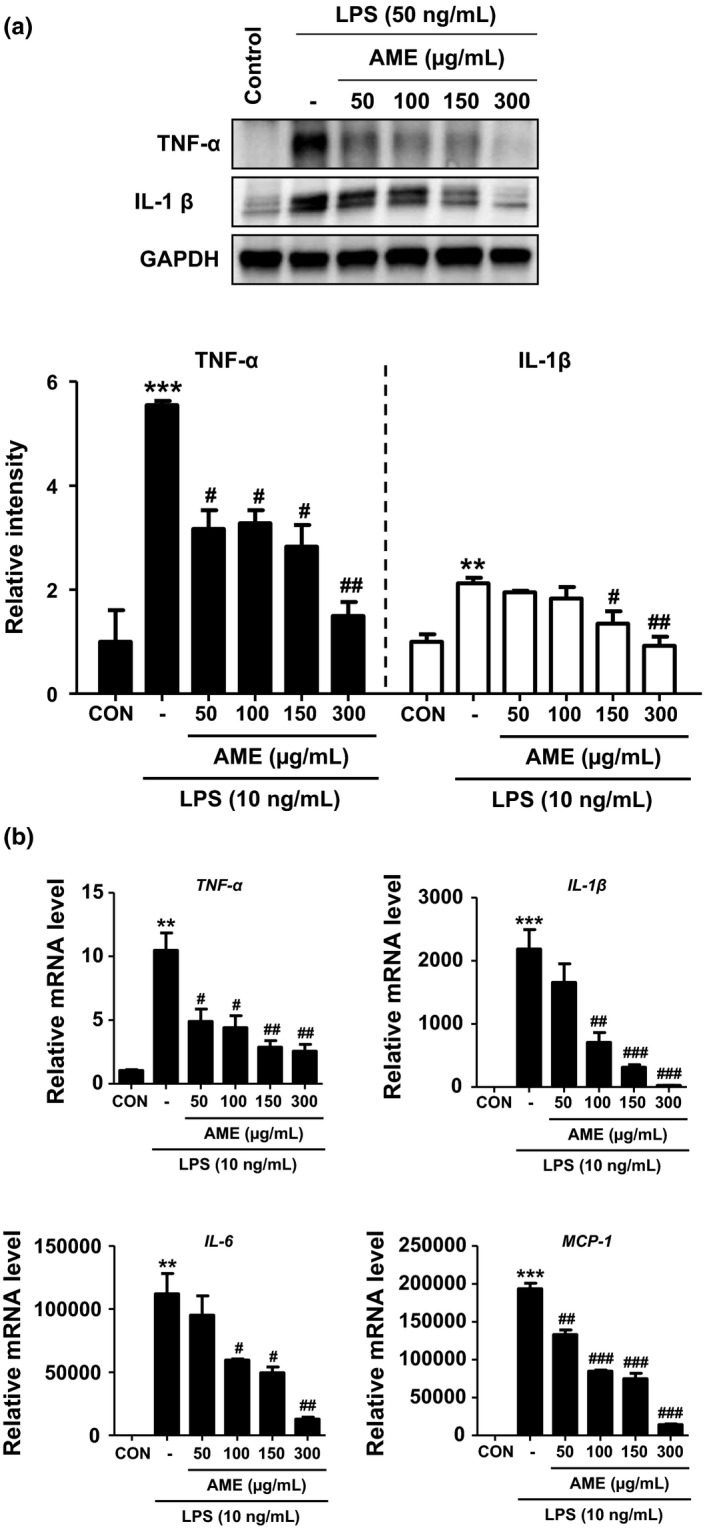
Inhibitory effects of *Antirrhinum majus* extract (AME) on lipopolysaccharide (LPS)‐stimulated pro‐inflammatory cytokine expression in RAW 264.7 cells. RAW 264.7 cells were treated with AME for 4 hr followed by LPS treatment (10 ng/ml) for 24 hr. (a) The pro‐inflammatory cytokine protein level was measured by Western blotting. The experiments were repeated at least three times, and representative Western blot images and quantification data are shown. (b) mRNA levels for inflammatory genes, as measured by RT‐qPCR in RAW 264.7 cells. **p < .01, ***p < .01 versus CON; ^#^ *p* < .05, ^##^ *p* < .05, ^###^ *p* < .05 versus LPS treatment only

Macrophages secrete proteins called cytokines, which are signaling molecules that react with receptors on the cell surface to trigger certain reactions. LPS stimulates macrophages to secrete cytokines that typically play a central role in an inflammatory response, such as TNF‐α, IL‐6, IL‐1β, and MCP‐1. Therefore, Western blot analysis was performed to examine the inhibitory effects of AME on the expression of cytokines in RAW 264.7 cells activated by LPS (Figure 5a). The expression of TNF‐α and IL‐1β increased considerably in the group with RAW 264.7 cells treated with LPS, compared to that in the control group. However, the expression of TNF‐α and IL‐1β was significantly inhibited in the AME‐treated and LPS‐stimulated group. Moreover, by treating RAW 264.7 cells with AME, the expression of mRNA of pro‐inflammatory cytokines, *TNF‐α*, *IL‐6*, *IL‐1β*, and *MCP‐1*, was also found to be inhibited. Thus, compared to the control group that was treated with LPS only, the AME‐treated group showed similar inhibitory pattern for protein and mRNA expression of the studied inflammatory cytokines.

*Antirrhinum majus*, a plant with the edible flowers used as a food item in Korea, has attracted attention as a rich source of phytochemicals, which are beneficial for human health. It has been used as an alternative therapy and traditional medicine to treat various diseases. However, its biological properties have not been fully elucidated. Therefore, in the present study, we investigated the effects of flavonoids involved in Korean AM. AME prevented the induction of iNOS and COX‐2 by LPS in RAW 264.7 macrophages. Furthermore, AME suppressed both the LPS‐induced production of pro‐inflammatory cytokines, at the mRNA and protein level, in macrophages. Although the anti‐inflammatory effects of AME are known, this is the first report demonstrating that AME inhibits inflammation in macrophages.

Flavonoids are widely existed in the plants and various biological functions in previous studies (Kumar & Pandey, 2013; Lopes, Schulman, & Hermes‐Lima, 1999). These studies suggest that flavonoids have various pharmacological properties such as anti‐cancer, antioxidant, antihypertensive, antiatherosclerotic, and anti‐inflammatory properties (Lee et al., 2012; Ray et al., 1999; Wang et al., 1999). Of these biological properties, the anti‐inflammatory effects of flavonoids have used for a long time. Inflammation is the normal physiological response to a variety of tissue injuries, chemical agents, and bacterial infection (Ariel, Maridonneau‐Parini, Rovere‐Querini, Levine, & Mühl, 2012; Khan et al., 2011; Liu, Zou, Chai, & Yao, 2014). Macrophages play a significant role in inflammation by overproducing pro‐inflammatory cytokines and inflammatory mediators (Chawla, Nguyen, & Goh, 2011; Laskin, 2009; Wynn, Chawla, & Pollard, 2013). Chronic inflammation causes the increase of pro‐inflammatory mediators, including iNOS and COX‐2, and various cytokines, including MCP‐1, TNF‐α, IL‐6, and IL‐1β (Gabay, 2006; Kushner, 1993). NO, synthesized by iNOS, has been recognized as important inflammatory mediator and a mediator of cell communication in macrophages (Wang et al., 1999; Wojdasiewicz et al., 2014). iNOS is expressed after exposure to specific stimulants such as LPS and cytokines (Wong et al., 1986). Thus, the regulation of iNOS and COX‐2 is important in the inflammation response.

## 4 CONCLUSIONS

In this study, an ethanol extract of AM was prepared and its antioxidative and anti‐inflammatory properties were investigated. AME showed high polyphenolic and flavonoid contents as well as high DPPH and ABTS radical‐scavenging ability. To test the cytotoxicity of AME, the viability of RAW 264.7 macrophages was assessed. The anti‐inflammatory effects of AME were investigated via inhibition of NO production and changes in expression of inflammation‐related proteins and mRNA in RAW 264.7 cells activated by LPS stimulation. Exposure of RAW 264.7 macrophages to LPS was associated with an accumulation of NO in the culture medium, suggesting an enhanced NO production. This LPS‐induced NO production by Raw 264.7 macrophages was inhibited by AME, in a concentration‐dependent manner. Furthermore, AME inhibited iNOS and COX‐2 protein expression in a concentration‐dependent manner in LPS‐stimulated Raw 264.7 macrophages. Investigation of inhibitory effects on expression of cytokines that play a central role in inflammatory stage showed that AME treatment inhibited the LPS‐induced expression of TNF‐α and IL‐1β protein expression as well as the mRNA levels of *TNF‐α*, *IL‐6*, *IL‐1β*, and *MCP‐1* in Raw 264.7 macrophages in a concentration‐dependent manner. Collectively, these findings indicate that AME showed anti‐inflammatory effects in macrophages. Although more detailed research is needed to obtain further insights into the exact anti‐inflammatory mechanism of AME, the findings of our study may serve as important basic data for studies on anti‐inflammatory substances via extraction of active ingredients from AM, as well as studies on isolation and mechanism of action of inflammation‐inhibiting components for prevention or treatment purposes.

## CONFLICT OF INTEREST

The authors declare that they do not have any conflict of interest.

## ETHICAL APPROVAL

This study does not involve any human or animal testing.
